# Timing of Meals and Exercise Affects Hormonal Control of Glucoregulation, Insulin Resistance, Substrate Metabolism, and Gastrointestinal Hormones, but Has Little Effect on Appetite in Postmenopausal Women

**DOI:** 10.3390/nu13124342

**Published:** 2021-12-01

**Authors:** Katarina T. Borer, Po-Ju Lin, Elizabeth Wuorinen

**Affiliations:** 1 School of Kinesiology, The University of Michigan, Ann Arbor, MI 48109, USA; Po-Ju_Lin@urmc.rochester.edu (P.-J.L.); ewuorine@nmu.edu (E.W.); 2 Department of Surgery, University of Rochester Medical School, Rochester, NY 14642, USA; 3 School of Health and Human Performance, Northern Michigan University, Marquette, MI 49855, USA

**Keywords:** timing of meals and exercise appetite assessment, glucose, insulin, HOMA-IR, glucagon, CCK, GIP, GLP-1, ghrelin, PYY

## Abstract

The current prevalence of obesity in the US is strongly associated with excessive food intake and insufficient physical activity. This study examined whether changing the timing of exercise before or after two daily meals could alter human appetite for food. Fifty-four healthy postmenopausal women were matched by body weight and assigned to two groups: (1) two bouts of 2-h moderate-intensity exercise ending one hour before each weight-maintenance meal (XM, *n* = 23), (2) two-hour moderate-intensity exercise starting 1 h after each weight-maintenance meal (MX, *n* = 23), and one sedentary control (SED) arm (*n* = 8). Measurements included appetite ratings, circulating glucose, free fatty acids (FFAs), a ketone body D-ß-hydroxybutyrate (BHB), glucoregulatory hormones insulin and glucagon, and gastrointestinal hormones associated with food digestion and absorption and implicated in appetite sensations. XM group increased concentrations of FFAs and BHB during exercise and increased insulin and homeostatic assessment of insulin resistance (HOMA-IR) during postprandial periods. MX group reduced postprandial insulin and HOMA-IR by about 50% without a major change in plasma glucose. There was brief suppression of hunger and an increase in satiation in both exercise groups near the end of the first postprandial period. The time course of hunger was unrelated to the perturbations in fuel metabolism, depletion of liver glycogen, and not correlated with concentration changes in hunger-stimulating hormone ghrelin during XM exercise before meals. Similarly, there was no correlation between the time course of fullness during exercise after meals with the postprandial secretion of gastrointestinal hormones including cholecystokinin (CCK) that has been linked to satiation. Hunger and satiation appear to depend on oral intake and gastrointestinal processing of nutrients and are not affected by metabolic and hormonal consequences of the timing of exercise with respect to meals. Moderate-intensity exercise performed shortly after meals induces a rapid and highly effective lowering of insulin resistance.

## 1. Introduction

It is important to understand physiological, hormonal, and metabolic influences on hunger and satiation linked to meal eating and physical activity to better provide effective solutions for weight management. In the US, the majority of residents engage in comparatively low levels of physical activity [1] and in excessive nutrient intake [2]. According to current CDC reports, 42.4% and 31.1% of Americans were obese and overweight, respectively in 2018 [3,4]. The high prevalence of excess weight causes psychological, health, and financial burdens for individuals, community, society, and has economic impacts. Daily meal eating and physical activity are intermittent human behaviors that are largely under voluntary control, but also are responsive to body energy status. These two behaviors affect body energy balance in opposite ways. Feeding adds to positive energy balance assisted by gastrointestinal hormones and insulin, while physical activity expends energy and stored metabolic fuels assisted by systemic catabolic hormones. Our body weight and body fat reflect choices of what, when, how much we eat and our activity level. 

There still are conflicting points of view regarding the origin of signals that guide our appetite. Theoretical and research interests have ranged from examining whether the gastrointestinal tract itself provides cues regarding the level of hunger and satiation [5], whether hormones and metabolites during fasting, food consumption, or exercise signal the state of nutrition, or whether guiding signals arise from the state of depletion or repletion of the body energy stores. Among gastrointestinal signals, classic experiments singled out hunger pangs as the origin of hunger [5] and the sensation of stomach fullness as satiation [6,7]. The term satiation therefore indicates the positive sensation at the termination of a meal [8] and is used here interchangeably with the term stomach fullness. Other hypotheses attribute hunger and satiation to metabolites and hormones elicited in different prandial states that reflect acute energy status and influence brain appetite and reward centers. Blood glucose concentration received much attention because of its role as a principal brain fuel [9]. Blood glucose availability is limited to a small 3000 kcal store of liver glycogen and a limited hepatic capacity for its synthesis [10] compared to a body fat depot typically greater than 100,000 kcal [10,11]. A decline in blood glucose concentration during fasting or after prolonged glycogen-depleting exercise suggests hypoglycemia as a possible signal for hunger shown by increased requests for food [12]. On the other hand, exercise energy expenditure and its hormonal and metabolic sequelae have not been consistently associated with hunger, and at higher intensities have been reported to suppress the appetite [13]. Ghrelin is considered as a hunger signal, in part because of the similarity in the time course of its concentration changes and the development of hunger [14], and in part because infusion of a supraphysiological concentration of this peptide into sated volunteers elicited hunger [15]. 

There has been greater support for the sensation of fullness or satiation responding to signaling between the gastrointestinal peptides and hormones with brain centers that control nutrient digestion and absorption [16,17]. A key hormone convincingly implicated in satiation is duodenal hormone cholecystokinin (CCK) [18]. Its role also is to aid in the digestion of fats and protein. Glucagon-like peptide-1 (GLP-1) [19] and peptide YY (PYY) [20] signal satiety [21], a reduced urge to eat after completion of a meal [8], They are released from the distal ileum and colon 2–3 h after eating [22]. Through their action as an ileal brake [23], they slow stomach emptying rate to reduce the arrival of partially absorbed excess nutrients from large meals to the distal intestine. They more likely serve as a feedback to food overconsumption rather than as satiation signals terminating normal-size meals. Signals from body fuel stores that appear to influence appetite include D-ß-hydroxybutyrate (BHB) and leptin. BHB is a ketone body signaling hepatic glycogen depletion after prolonged moderate-intensity exercise [24,25] when the liver begins oxidizing free fatty acids (FFAs) into ketones to provide an alternate brain fuel [26]. A better known and more influential hypothesis is that changes in the size of subcutaneous fat stores send a hormonal signal to the brain circuits controlling hunger and food consumption. The messenger is attributed to the hormone leptin the concentration of which declines during negative energy balance. The evidence that leptin can act as a powerful appetite suppressor is the basis for the homeostatic hypothesis of energy regulation [27]. The hypothesis derives from leptin’s ability to reduce hunger and cause loss of body fat in morbidly obese individuals with congenital leptin deficiency [28]. The homeostatic hypothesis calls for leptin to elicit hunger when both its concentration and the mass of subcutaneous adipose tissue decline and to exert a negative feedback over hunger when its concentration and the mass of adipose tissue increase. However, the hypothesis that leptin controls both long-term energy balance and hunger and satiation in meal-to meal eating in healthy individuals has not received support. In a large trial with overweight individuals [29], leptin administration ranging from the physiological to supra-physiological concentrations failed to affect body fat or food intake and validate leptin’s negative feedback role.

Two additional factors add to the complexity of issues regarding how daily hunger and satiation are controlled. The first is that hunger is under circadian control. Ratings of daytime hunger are lowest in the morning despite 8 to 10 h of overnight fasting and are at the peak level in the afternoon [30]. Likewise, leptin also displays a circadian pattern with the highest concentrations during the night, and lowest during the daytime [31]. The second factor that requires consideration is that human stomach size adapts to varying levels of food consumption, and therefore its fullness signals may be attenuated if the stomach size increases beyond its usual size of a grapefruit. Individuals who engage in food binging have a larger stomach volume than individuals who do not, and the effect is related to binge-eating and not to their body weight [32]. This effect is amplified in people engaging in hot-dog eating competitions. They develop up to 700% greater capacity to eat rapidly and store greater quantities of food in their stomachs [33]. The anatomical changes associated with binging, gorging and purging, have recently been found to affect brain circuits mediating hunger and satiation [34]. Conversely, a prolonged year-long total fast that resulted in massive weight loss was reported to produce no substantial weight-regain rebound 5 years later [35] suggesting appetite control through stomach atrophy due to disuse.

This brief review of the variables influencing human appetite suggests that control of hunger and satiation in daily meal-to-meal eating is still not fully understood, and a systematic re-examination is warranted. We were particularly interested in whether the timing of eating and exercise could be used to harness the antagonistic effects of hormones and metabolites on hunger and satiation elicited by eating on one hand, and by performing moderate-intensity exercise, on the other, as the former serves to increase energy gain, and the latter to increase energy expenditure. To address this issue, we utilized a timing-of-meals-and-exercise experimental paradigm that allows an examination of the effects of close succession and alternation of antagonistic hormonal and metabolic effects of these two behaviors. By providing two daily weight-maintenance meals preceded by an hour, or followed by an hour, by 2-h moderate-intensity treadmill walking, one in the morning and the other in the afternoon, we also could examine whether circadian timing of these behaviors in the afternoon would produce a different effect compared to behaviors performed in the morning as was shown in studies of the timing of meals of different size on weight loss in obese women [36].

We hypothesized that the antagonistic effects of differential timing of exercise and meals would:

(1) Increase hunger when exercise was performed before meals;
(2) Decrease satiation, insulin and gut hormones when exercise was performed after meals;
(3) Produce different effects on hunger and satiation in the afternoon than in the morning.

## 2. Materials and Methods

### 2.1. Subjects

The 54 healthy postmenopausal subjects were recruited from the University of Michigan clinical studies webpage (UMClinicalStudies.org, accessed 8 June 2008) and local newspaper advertisements. They participated in two studies with the same timing-of-meals-and-exercise experimental design. The inclusion criteria were: 50 to 65 years old, surgical or natural menopause with no menstrual periods for at least one year, no metabolic disease, body mass index (BMI) of 24 to 30 kg/m^2^, no hormone replacement therapy, non-smoker, normal fasting glucose and insulin, hematocrit > 32%, hemoglobin > 12 mg/dL, absence of musculo-skeletal disabilities that would prevent treadmill walking, and sedentary status (<60 min of regular exercise per week). In the larger study, after matching for age and BMI, 13 subjects were assigned to exercise before the meals (XM), 13 subjects to exercise after the meals (MX), and 8 remained sedentary (SED). The results on 8 subjects in each exercise group and on the 8 sedentary subjects were reported earlier in a study examining the effects of different carbohydrate content of the meals [37]. In the smaller study, 20 subjects were similarly matched with 10 assigned to XM and the other 10 to MX treatment. The studies were conducted in accordance with the Declaration of Helsinki. The study protocol HUM0001787 was approved by the University of Michigan Medical School Institutional Review Board (IRB-MED) on 8/7/2008, and all subjects signed the informed subjects consent form approved by the IRB-MED. 

Table 1 presents demographic characteristics of the subjects, none of which differed between the groups with the exception of BMI. Also shown are energy costs of provided meals and of assigned exercise as they affected energy balance.

### 2.2. General Experimental Protocol 

Subjects underwent preliminary health and fitness screening at the Michigan Clinical Research Unit (MCRU). The health screen included health history, measurements of weight, height, BMI, and a blood draw for fasting glucose, fasting insulin, and baseline TSH as a check against possible hypothyroidism. In the larger study, body composition was assessed by a dual-energy X-ray (DXA) absorptiometry apparatus (model Prodigy, Lunar Radiation Corporation, Madison, WI, USA). In the smaller study, it was assessed by bio-impedance procedure (RJL Systems, Clinton, MI, USA). A fitness screen assessed individual maximal aerobic effort. It consisted of a treadmill test at 4.8 km/h speed with 2% slope increments every 3 min with the subject breathing through a mouthpiece using a Max II metabolic cart (AEI Technologies, Inc., Bastrop, TX, USA). The criterion of maximal effort was a respiratory quotient of 1 (calculated from the rate of carbon dioxide produced over the rate of oxygen consumed). 

### 2.3. Study Design

**Figure 1:**
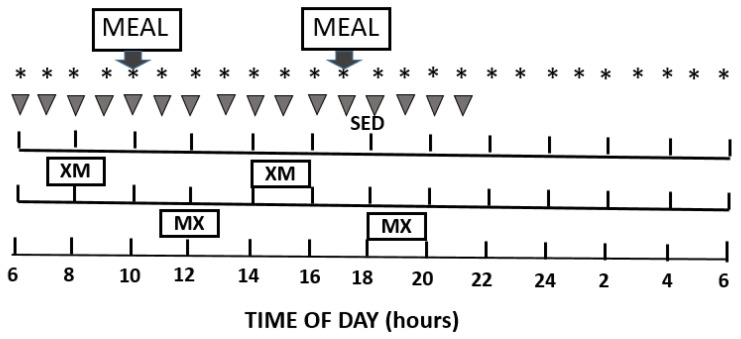
Study experimental design. Horizontal boxes show the sedentary group, timing of exercise before the meals (XM), exercise after the meals (MX) and the times of meals at 10:00 h and 17:00 h. Inverted triangles indicate times when the appetite measurements were taken on the visual analog scale (VAS). Asterisks (*) indicate hourly blood collection.

At 18:00 h, the evening before the study day (Figure 1), subjects were admitted to MCRU. A meal containing 10 kcal/kg body-weight was provided at 19:00 h. Hourly blood collection over 24 h was initiated at 06:00 h on the study day through an antecubital vein catheter kept patent with sodium heparin. Additional samples were taken at 15- and 30-min intervals during meals and exercise. Measured metabolites included glucose, free fatty acids (FFAs), and ketone body D-ß hydroxybutyrate (BHB). Measured hormones included insulin, glucagon, glucose-dependent insulinotropic polypeptide or gastric inhibitory polypeptide (GIP), glucose dependent polypeptide 1 (GLP-1), peptide YY (PYY), ghrelin, leptin, and cholecystokinin (CCK). In addition, psychophysical sensations of hunger, desire to eat, capacity to eat, and fullness were measured hourly, and at 30 min before the start of meals and exercise, using a validated visual analog scale (VAS) [38]. 

Timing of meals and exercise entailed 2 experimental conditions: 2-h moderate-intensity exercise twice in a day either completed 1 h before the 2 daily meals at 10:00 and 17:00 h (XM), or initiated 1 h after the meals (MX). XM exercise bouts were completed between 07:00 and 09:00 h and 14:00 and 16:00 h while the MX exercise bouts, were carried out between 11:00 and 13:00 h and 18:00 and 20:00 h. Resting metabolic rate (RMR) was measured between 06:00 and 06:30 h on the day of the study and the next morning, when subjects just woke up but were still laying on bed, with their head and face covered by a transparent canopy hood connected to the Viasys metabolic system (Respiratory Care Inc.,Yorba Linda, CA, USA). Exercise energy expenditure was measured with a metabolic cart during the first half of each hour of exercise. 

### 2.4. Exercise Intensity and Substrate Metabolism

Subjects walked at 45% of their maximal effort and at a constant treadmill speed of 4.8 km/h, with modifications of the treadmill incline to adjust the relative effort. Substrate metabolism was calculated using the equation [39] in which the metabolic quotient reflects metabolic fuel partitioning between carbohydrates and fat and allows for calculation of carbohydrate and fat utilization during exercise.

### 2.5. Meals

In addition to the pre-trial evening meal, two isocaloric weight-maintenance meals were provided at 10:00 h and 17:00 h on the trial day, each containing one half of the daily weight-maintenance energy (which was assessed as 25 kcal/kg body-weight). The meal macronutrient composition was 60% carbohydrate, 15% protein, and 25% fat recommended by Departments of Agriculture and Health and Human Services since 2010 [40]. Subjects were encouraged to eat meals within 30 min. Food provided and any left uneaten was weighed in the larger study to determine energy and nutrient consumption and allow comparisons to energy expended in exercise. Table 2 and Table 3 show the macronutrient composition and calories in the food eaten in two daily meals in all trials normalized for a subject weighing 64.5 kg.

### 2.6. Appetite Assessment

On the study day, subjects rated their appetite on a 100 mm VAS [38] every hour from 06:00 to 21:00 h and also at 30 min after they completed the meals (at 10:30 h and 17:30 h). The VAS questions: How hungry do you feel right now? How full do you feel right now? How strongly do you desire to eat right now?, and How much could you eat right now? required a mark on a scale bracketed with “Not at all” at one end and “Extremely” at the opposite end of the scale. The distances marked were converted to percentages of the full scale.

### 2.7. Blood Collection

Blood samples were collected hourly into ice-chilled EDTA-coated tubes containing aprotinin (50 KIU/mL blood, Sigma Chemical, St. Louis, MO, USA) and dipeptidyl peptidase-4 inhibitor (10 μL/mL blood; EMD Millipore Corporation, Billerica, MA, USA) to protect gut peptides from degradation. Plasma was kept frozen at −80 °C for hormone measurements. 

### 2.8. Hormone and Metabolite Measurements

The basal concentrations of TSH were measured by the University of Michigan Chemistry Laboratory as part of qualification for inclusion in the study. Metabolites glucose (Fisher Diagnostics, Middletown, VA, USA) and FFAs (Wako Diagnostics, Richmond, VA, USA) were measured in both studies. Measurement of ketone body Beta-3-hydroxybutyrate (BHB) was included in the larger study to serve as indicator of exercise-induced liver glycogen depletion [25,26]. Ketone body was measured with a kinetic enzymatic method (Randox Laboratories-US, Ltd., Kearneysville, WV, USA). Both studies measured plasma insulin, glucagon, and ghrelin. Insulin and glucagon were measured with radioimmunoassays (EMD Millipore Corporation, Billerica, MA, USA). The intra-assay coefficients of variation (CV) for the insulin and glucagon assays were respectively 2.3% and 3.6%, and inter-assay CVs were 16.2% for both assays. Total human ghrelin was measured with a radioimmunoassay kit from Phoenix Pharmaceuticals (Belmont, CA, USA) with a sensitivity ranging between 4.76 pg/tube at 80% binding and 14.9% at 50% binding. The larger study measured plasma leptin, ghrelin, GLP-1, GIP, PYY, and cortisol. Leptin was measured with an RIA kit (HL81HK, Linco Research, Millipore Corp, St Charles, MO, USA). The intra- and inter-assay CVs for leptin were 9.1 and 14.2%, respectively. Plasma GIP, GLP-1, and PYY were measured with a milliplex chemiluminescent assay kit (HGT-68K, EMD Millipore Corporation, Billerica, MA, USA). The intra-and inter-assay coefficients of variation (CV) for GIP, GLP-1, and PYY were <11% and <19%, respectively. Cortisol was measured with a solid-phase radioimmunoassay (Siemens Medical Solutions Diagnostics, Los Angeles, CA, USA). Intra- and inter-assay CVs were between 3% and 5.1%, and 4% and 6.4%, respectively. CCK was measured only in the smaller study with Euria-CCK radioimmunoassay (Alpco Diagnostics, Windham, NH, USA). The intra-assay and inter-assay CVs for CCK were 2.17 and 10.8%, respectively.

### 2.9. Statistical Analyses

Data are presented as means and standard errors of the means (SEMs). To eliminate inter-personal variability, hormones are presented as percent change from the 06:00 h concentration value. All statistical analyses were performed with SAS (Statistical Analysis Software, version 9.4, SAS Institute, Cary, NC, USA). Between-group comparisons of metabolite, hormone, and appetite values were performed with repeated-measures mixed-model ANOVA where the treatment and time and their interactions were analyzed as between-subject effects, and the values for each of 54 subjects, as within-subject random intercept. Where no overall between-group differences were seen, time slice effects indicated significant group differences at specific time periods. As in some instances this analysis suggested a difference in the magnitude of metabolic, hormonal, and psychophysical changes between the morning and afternoon exercise bouts and postprandial periods, an analysis of the effects of timing of the morning (AM) and afternoon (PM) exercise bouts and of the AM and PM postprandial periods was performed on the respective areas under the curve (AUCs) calculated using trapezoidal rule. In the exercise groups, the AUCs were 3 h long and included 2 h of exercise and 1 post-exercise hour until the start of the meal. XM exercise AUCs were between 07:00 h and 10:00 h and between 14:00 h and 17:00 h. MX exercise AUCs were between 11:00 h and 14:00 h and between 18:00 h and 21:00 h. The overall effect of the meals was measured with 7-h AM and PM postprandial AUCs. The morning postprandial AUC extended from the onset of the morning meal at 10:00 h until the onset of the afternoon meal at 17:00 h and included a 2-h XM exercise between 14:00 h and 16:00 h. The afternoon postprandial AUC extended for 7 h (17:00 h to 24:00 h) after the start of the PM meal and did not include an exercise period. For all but four outcome variables, overall between-group differences were calculated for the 3-h XM and MX exercise AUCs from measurements at their assigned time of exercise using mixed-model ANOVA for between-group AM-PM difference scores. However, glucose, insulin, HOMA-IR, and ghrelin showed no group differences during their assigned XM exercise times when MX and SED groups were not exercising. Instead, the treatment effects of XM exercise were manifested during the postprandial period at the time period assigned to MX exercise. For that reason, AUC timing analyses in XM, MX and SED trials for glucose, insulin, HOMA-IR, and ghrelin were carried out both at the assigned XM treatment time when XM exercise exhibited no treatment effects, and at the 3-h postprandial period assigned for MX exercise when the XM treatment effects and meal MX meal and exercise effects were observed. Therefore the bar diagrams presenting AUC analyses for glucose, insulin, HOMA-IR, and ghrelin have two rows of designations in their abscisse. The top row designation identifies the treatment group, and the bottom designation identifies the time period (XM or MX) for which the analyses were performed. Within-group comparisons of differences between both exercise and postprandial AM and PM AUCs, were calculated with the paired t test procedure using Tukey-Kramer adjustment for multiple comparisons. Insulin resistance was assessed with the homeostatic model (HOMA-IR) test [41] during both the fasting and the postprandial period. As isocaloric meals of the same macronutrient composition were used in this study, postprandial HOMA-IR was estimated for meal tolerance, the procedure [42] validated against the minimal model and the intravenous glucose tolerance test [43]. To calculate HOMA-IR, the product of insulin and glucose AUCs was divided by 405. Figure graphics were produced with GraphPad Prism 8.4.2 software (GraphPad Software Inc., San Diego, CA, USA).

## 3. Results

### 3.1. Exercise Parameters 

Walking speed, distance traveled on the treadmill, and total calories expended during either morning or afternoon exercise did not differ between the XM and MX exercise groups (Table 4). Using the metabolic energy expenditure equivalents (METs) for exercise which represent multiples of resting metabolic rate per hour, mean XM energy expenditure was 3.9 MET, and MX energy expenditure was 4.5 MET. Fat was utilized more than carbohydrate during morning XM, compared to MX exercise (57% vs. 37%), and carbohydrate substrate was utilized more during the morning MX exercise than XM exercise (63% vs. 43%). In the afternoon, carbohydrates were the predominant fuel utilized in both exercise trials with 60% of total energy used in the XM, and 67% in the MX trial.

### 3.2. Exercise- and Meal-Timing Effects on Circulating Metabolic Fuels

#### 3.2.1. Exercise- and Meal-Timing Effects on Free Fatty Acids (FFAs) 

**Figure 2:**
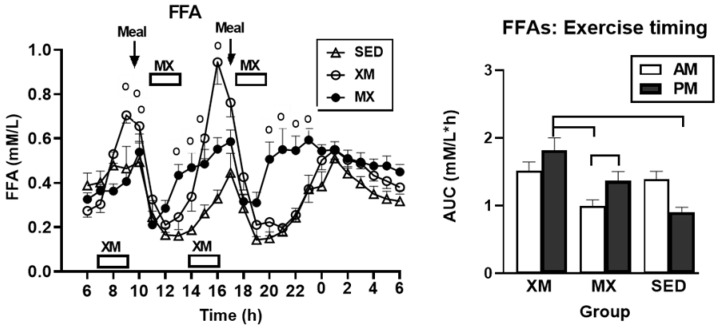
Exercise- and meal-timing effects on the concentrations of FFAs and their AUCs. Circle superscripts identify time slices when group concentration differences were significantly different (**left** panel). AUCs represent 2 h of exercise and one post-exercise hour. Afternoon XM AUCs were significantly higher than morning MX and afternoon SED AUCs. Within the MX trial, PM AUC was higher than the AM AUC (**right** panel). Brackets identify groups with significant between- and within-group AUC differences (**right** panel). XM = exercise before meals, MX = exercise after meals, SED = sedentary group.

For FFAs, only time (F_(df=54/2807)_ = 27.90, *p* < 0.0001) and treatment-time interaction effects were significant (F_(df=108/2807)_ = 8.23, *p* < 0.0001). The time slices, when group differences were significant (Figure 2, left panel), occurred during XM exercise between 08:30 h and 09:30 h in the morning trial (F_(df=2/233)_ = 3.8 to 24, *p* = 0.024 to <0.0001) and between 14:30 h and 17:00 h during the afternoon trial (F_(df=2/233)_ = 3.6 to 23.8, *p* = 0.029 to <0.0001). During MX exercise, time slices were significant at 13:30 h to 14:00 h after the morning trial (F_(df=2/233)_ = 10 to 13.5, *p =* 0.0024 to <0.0001) as well as at 20:00 h to 23:00 h after the afternoon exercise (F_(df=2/233)_ = 3.4 to 13.7, *p* = 0.034 to <0.0001).

For FFAs, only the 3-h exercise AUCs, but not 7-h postprandial AUCs, differed between and within groups (F_(df=5/75.8)_ = 6.83, *p* < 0.0001, Figure 2, right). XM afternoon AUCs were higher than morning MX AUCs (t_(df=71.9)_ = 4.39, *p* = 0.0005) and afternoon SED AUCs (t_(df=71.9)_ = 3.49, *p* = 0.0102). PM AUCs were higher than the AM AUCs within the MX trial (t_(df=51)_ = 3.30, *p* = 0.0178). 

#### 3.2.2. Exercise- and Meal-Timing Effects on D-ß-Hydroxybutyrate (BHB) Concentrations 

**Figure 3:**
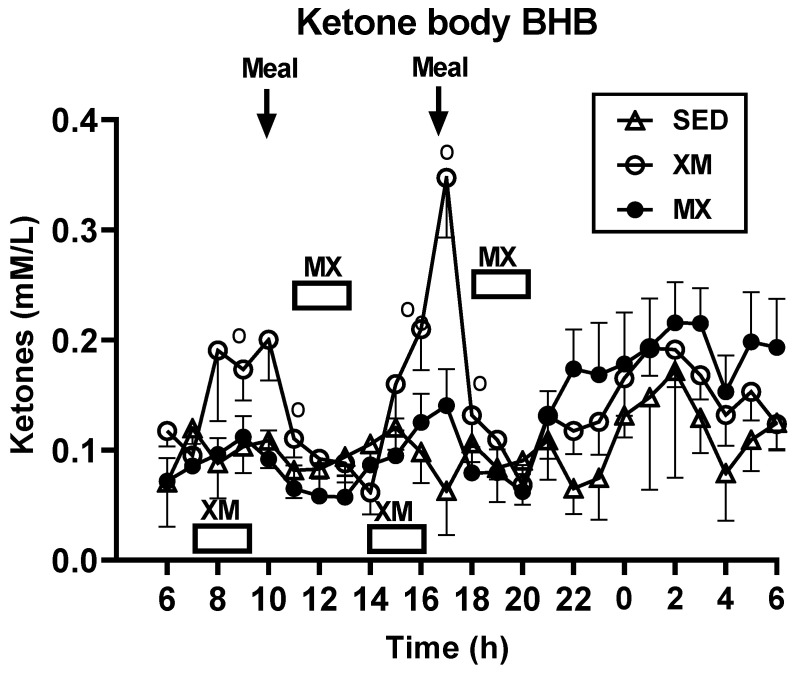
Exercise- and meal-timing effects on BHB concentrations. Circle superscripts identify time slices when group differences were significantly different. XM = exercise before meals, MX = exercise after meals, SED = sedentary group.

As was the case for FFAs, for BHB, only time (F_(df=53/1326)_ = 5.21, *p* < 0.0001) and treatment-time interaction effects (F_(df=93/1325)_ = 2.71, *p* < 0.0001) were significant. Group differences (Figure 3) were significant at time slices during the two XM exercise bouts, between 09:30 h and 10:00 h in the morning (F_(df=2/261)_ = 2.69 to 9.38, *p* = 0. 05 to <0.0001) and between 16:30 h and 17:45 in the afternoon (F_(df=2/278)_ = 6.0 to 17.45, *p* = 0.0029 to <0.0001).

**Figure 4:**
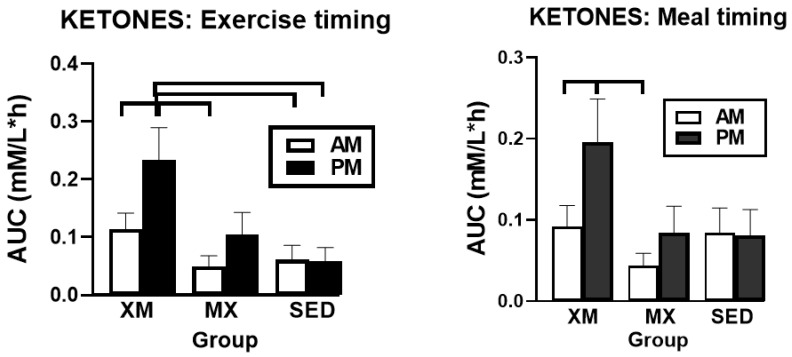
Exercise- and meal-timing effects on BHB exercise and prandial AUCs. Exercise-timing AUCs represent 2 h of exercise and one post-exercise hour. Meal-timing AUCs represent 7 postprandial hours.In the exercise analysis, afternoon XM ketone AUC was significantly higher than AM MX and both AM and PM SED AUCs. Within XM trial, afternoon AUC was higher than the AM AUC (**left** panel). In the prandial analysis, afternoon XM ketone AUC was significantly higher than AM MX AUC and the AM AUC within its own trial (**right** panel). Brackets identify groups with significant between- and within-group differences. XM = exercise before meals, MX = exercise after meals, SED = sedentary group.

Between-group differences in 3-h exercise AUC analysis (F_(df=5/45.3)_ = 4.61, *p* = 0.0017) included higher PM AUCs in the XM group than AM AUCs in MX group (t_(df=47)_ = 3.73, *p* = 0.0066) and both morning (t_(df=47)_ = 3.07, *p* = 0.04) and afternoon (t_(df=47)_ = 3.11, *p* = 0.036) SED AUCs (Figure 4, left panel). PM AUCs were higher than AM ones within the XM trial (t_(df=31)_ = 3.69, *p* = 0.0075). In the 7-h postprandial AUC analyses (F_(df=5/45.3)_ = 3.05, *p* = 0.0188, right panel), PM values in the XM trial were higher than AM values of the MX group (t_(df=50)_ = 3.29, *p* = 0.0224) and AM values within the XM trial (t_(df=31)_ = 3.12, *p* = 0.035).

#### 3.2.3. Exercise- and Meal-Timing Effects on Glucose Concentrations and AUCs 

**Figure 5:**
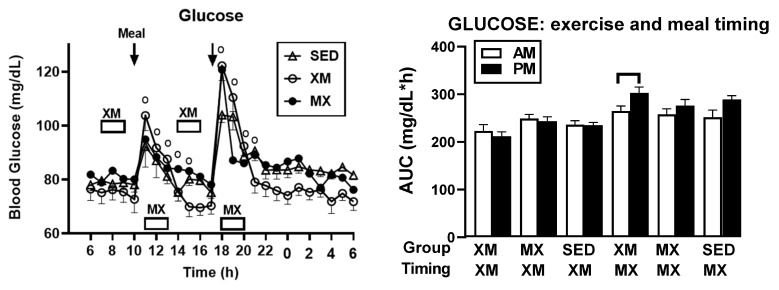
Exercise- and meal-timing effects on glucose concentrations (**left** panel) and AUCs (**right** panel). Circle superscripts identify time slices when group differences were significantly different (**left** panel). Timing analysis of group AUCs (treatment indicated in the top abscissa row) is presented for time periods assigned to XM and MX exercise (indicated in the bottom abscissa row). Bracket (**right** panel) identifies asignificant within-group difference in postprandial timing of XM trial. XM = exercise before meals, MX = exercise after meals, SED = sedentary group.

Glucose concentration revealed no overall treatment effect, but showed significant time (F_(df=54/2808)_ = 31.13, *p* < 0.001) and treatment-time interaction effects (F_(df=108/2808)_ = 4.29, *p* < 0.001). Significant group differences (Figure 5, left panel) occurred during the postprandial time slices at 11:15 h to15:30 h in the morning and early afternoon (F_(df=2/154)_ = 3.02 to 5.52 and *p* = 0. 05 to 0.0048), and at 18:00 h to19:00 h in the late afternoon (F_(df=2/278)_ = 3.52 to 14.93 and *p* = 0.032 to <0.0001). The lowest glucose concentrations at about 70 mg/dL occurred during the afternoon XM exercise bout between 15:00 h and 17:00 h.

No between-group glucose AUC differences were seen during the 3-h XM exercise period (marked by lower abscissa row, right panel). Between-group differences (indicated in the upper abscissa row in the right side of the right panel) were significant (F_(df=5)_ = 6.72, *p* < 0.0001) when AUCs were analyzed during the 3-h postprandial time period assigned to MX exercise (lower abscissa row, right panel). The source of the significance was a higher postprandial PM than AM AUCs within the XM trial (t_(df=21)_ = 4.35, *p* < 0.0001).

### 3.3. Exercise- and Meal-Timing Effects on Hormonal Control of Glucoregulation

#### 3.3.1. Exercise- and Meal-Timing Effects on Insulin 

**Figure 6:**
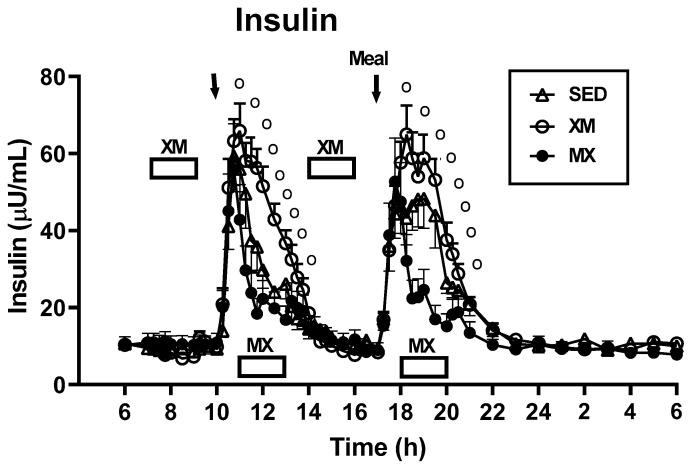
Exercise- and meal-timing effects on insulin concentrations. Circle superscripts identify time slices when group differences were significantly different. XM = exercise before meals, MX = exercise after meals, SED = sedentary group.

For insulin concentration, overall effects of treatment (F_(df=2/51)_ = 6.67, *p* < 0.0027), time (F_(df=54/2808)_ = 62.63, *p* < 0.0001), and treatment-time interaction (F_(df=108/2808)_ = 6.90, *p* < 0.001, Figure 6), were all significant (Figure 6). Significant group differences resulting from both morning and afternoon MX exercise were manifested only during the postprandial period as indicated by significance slices between 10:30 h and 13:30 h (F_(df=2/547_ = 3.922 to 37.11 and *p* = 0.0205 to <0.0001) and between 18:00 h and 20:00 h (F_(df=2/547)_ = 5.16 to 37.5 and *p* = 0.006 to <0.0001), respectively.

**Figure 7:**
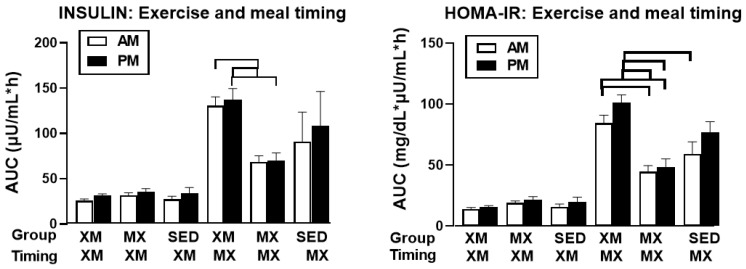
Exercise- and meal-timing effects on insulin AUCs (**left**) and HOMA-IR AUCs (**right**). Both the morning and afternoon XM insulin AUCs were higher than the AM MX AUC. Afternoon MX insulin UC also was significantly higher than the PM MX AUC (**left** panel). Both the morning and afternoon XM HOMA-IR AUCs were significantly higher than the AM and PM MX AUCs. Evening XM HOMA-IR AUC also was significantly higher than the AM SED AUC (**right** panel). Brackets identify groups with significant between- and within-group differences. EX = exercise, XM = exercise before meals, MX = exercise after meals, SED = sedentary group.

No 3-h group differences were seen (identified by upper abscissa row)for insulin AUCs when the analysis was performed during the AM and PM periods assigned to XM exercise (marked in bottom abscissa row, left panel of Figure 7). Treatment group differences were significant (F_(df=5)_ = 6.08, *p* < 0.0001) when AUC timing analysis was performed during the 3-h postprandial period assigned to the MX exercise (indicated by lower abscissa row, left panel). Morning insulin XM AUCs were 47 and 46% higher than the respective AM (t_(df=67.9)_ = 4.71, *p* < 0.0001) and PM (t_(df=67.9)_ = 4.58, *p* < 0.0001) MX AUCs timed during the postprandial 3-h periods assigned to MX exercise. Similarly, afternoon insulin XM AUCs were 50 and 49% higher than the respective AM (t_(df=67.9)_ = 5.19, *p* < 0.0001) and PM MX AUCs during the postprandial periods (t_(df=67.9)_ = 5.07, *p* < 0.0001, right side of left panel). Postprandial insulin MX AUCs were 24 and 35% lower than the respective AM and PM sedentary AUCs, but the differences were not significant. 

#### 3.3.2. Exercise- and Meal-Timing Effects on HOMA-IR Measure of Insulin Resistance

No group differences (indicated in the top abscissa row Figure 7, right panle) were seen when 3-h HOMA-IR AUCs were measured during the AM and PM periods assigned to XM exercise (indicated by the lower abscissa row, right panel). Treatment differences among groups were significant (F_(df=5)_ = 7.65, *p* < 0.0001) when timing analysis was performed during the 3-h postprandial period assigned to MX exercise (indicated by lower abscissa row, right panel). Postprandial HOMA-IR XM AUCs in the morning were 47 and 42% higher than the respective AM (t_(df=73)_ = 4.07, *p* = 0.0001) and PM (t_(df=73)_ = 3.66, *p* = 0.0005) MX AUCs. Afternoon HOMA-IR XM AUCs were 56 and 52% higher than the respective AM (t_(df=73)_ = 5.78, *p* < 0.0001) and PM (t_(df=73)_ = 5.37, *p* < 0.0001) MX AUCs during the 3-h postprandial periods assigned to MX exercise. While HOMA-IR XM AUCs were 43 and 37% higher than the respective AM and PM SED AUCs, the only significant difference was between PM XM and AM SED AUCs (t_(df=73)_ = 3.08, *p* < 0.0029).

#### 3.3.3. Exercise- and Meal-Timing Effects on Glucagon 

**Figure 8:**
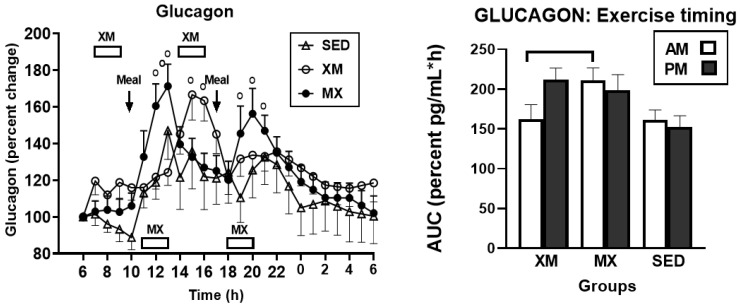
Exercise- and meal-timing effects on glucagon concentrations and its AUCs. Circles identify time slices when group differences were significantly different (**left** panel). Bracket (**right** panel) identifies a significant AUC difference between morning MX AUC being higher than morning XM AUC. EX = exercise, XM = exercise before meals, MX = exercise after meals, SED = sedentary group.

For glucagon, only the effects of time (F_(df=36/1441)_ = 8.38, *p* < 0.0001) and of treatment-time interaction (F_(df=66/1440)_ = 2.46, *p* < 0.0001 were significant (Figure 8, left panel). During three exercise-associated glucagon peaks, significant time slices were seen during the first morning MX exercise at 12:00 h to 13:00 h (F_(df=2/167)_ = 3.57 to 6.05, *p* = 0. 031 to <0.0029), during afternoon XM exercise at 15:00 h to 16:00 h (F_(df=2/167)_ = 3.47 to 4.07, *p* = 0. 0335 to 0.0183), and during afternoon MX exercise at 19:00 h to 20:00 h (F_(df=2/167)_ = 3.19 to 3.87, *p* = 0.023 and 0.044). Morning glucagon AUCs in MX group were significantly higher than the morning AUCs in the XM group (t_(df=31)_ = 4.60, *p* < 0.0001, right panel).

### 3.4. Exercise- and Meal-Timing Effects on VAS Appetite Ratings 

**Figure 9:**
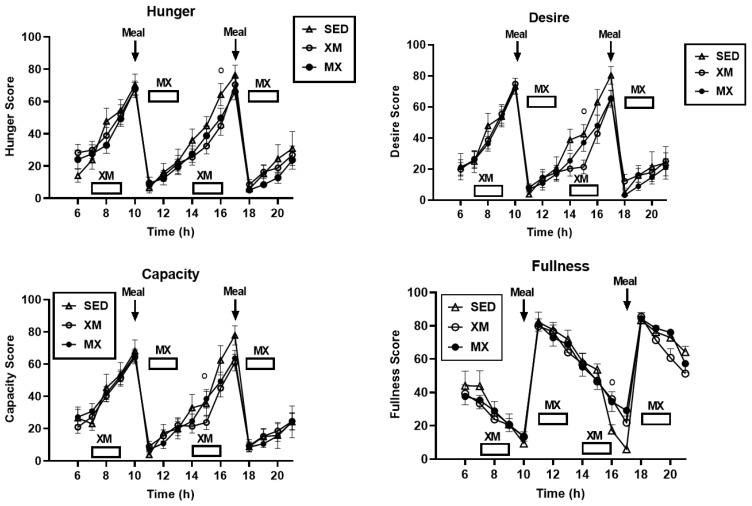
Visual analog scale ratings of hunger, desire to eat, capacity to eat, and fullness. Circles identify time slices when group differences were significantly different. XM = exercise before meals, MX = exercise after meals, SED = sedentary group.

VAS ratings revealed significant effects of time for desire (F_(df=23/897)_ = 9.63, *p* < 0.0001), capacity to eat (F_(df=22/901)_ = 8.38, *p* < 0.0001), and fullness (F_(df=23/899)_ = 19.19, *p* < 0.0001), but not for hunger (Figure 9). There were no significant treatment effects or treatment-time interactions for any of the four appetite measures. However, a single significant group difference for each of the four appetite measures occurred at 15:00 h time slice for hunger (F_(df=2/943)_ = 13.56, *p* < 0.0001), at 16:00 h for desire to eat (F_(df=2/319)_ = 4.44, *p* = 0.0125), and at 15:00 h for both capacity to eat (F_(df=2/886)_ = 11.43, *p* < 0.0001) and for fullness (F_(df=2/749)_ = 5.88, *p* = 0.0029). These slice effects were at the end, or immediately following the second XM exercise bout. Two exercise groups showed depressed values relative to SED group for hunger, and higher values relative to SED group for fullness. Slice effects for desire and capacity to eat did not show consistent treatment difference between exercising and SED trials.

**Figure 10:**
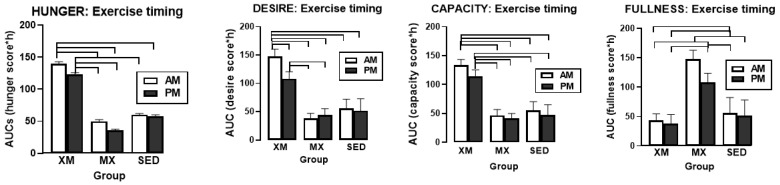
Exercise- and meal-timing effects on the VAS appetite score AUCs. Brackets identify groups with significant between- and within-group differences. Morning and evening XM hunger, desire, and capacity AUCs were significantly higher than MX and SED AUCs. Morning and afternoon MX AUCs were significantly higher than XM and SED AUCs. EX = exercise, XM = exercise before meals, MX = exercise after meals, SED = sedentary group.

Three-hour exercise AUC analyses performed at the respective exercise times were significantly different for all four appetite ratings (Figure 10, F_(df=5/75.8)_ = 9.82 for hunger, F = 10.43 for desire, F = 9.72 for capacity, and F = 10.41 for fullness, all at *p* < 0.0001), but not for 7-h postprandial period analyses. All between-group AUC differences exhibited t values (df = 74.3 between 2.79 and 6.33 and *p* values between 0.015 and <0.0001). A single difference between AM and PM AUCs was recorded in the XM trial for desire to eat (t_(df=51)_ = 3.81, *p* = 0.0004).

### 3.5. Exercise- and Meal-Timing Effects on Gastro-Intestinal Hormones GIP, GLP-1, PYY, Ghrelin, Leptin, and CCK

#### 3.5.1. Exercise- and Meal-Timing Effects on GIP, GLP-1, and PYY 

**Figure 11:**
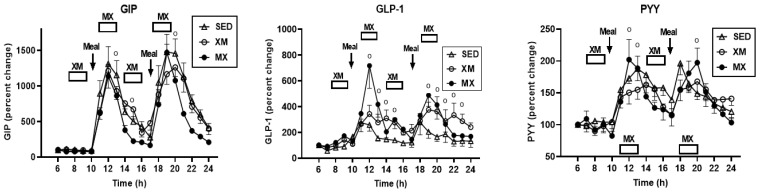
Exercise- and meal-timing effects on GIP, GLP-1, and PYY concentrations. Circle superscripts identify time slices when group differences were significantly different. XM = exercise before meals, MX = exercise after meals, SED = sedentary group.

Significant treatment effect was seen only for GLP-1 (F_(df=2/21)_ = 5.22, *p* = 0.0145) with the values for both exercise groups exceeding those of SED group (Figure 11, center). GIP, GLP-1, and PYY had significant effects for time (GIP: F_(df=20/280)_ = 31.36, *p* < 0.0001; GLP-1: F_(df=23/439)_ = 11.39, *p* < 0.0001, PYY: F_(df=22/440)_ = 8.17, *p* < 0.0001) and treatment-time interaction (GIP: F_(df=20/280)_ = 2.28, *p* = 0.0017; GLP-1: F_(df=23/436)_ = 1.94, *p* = 0.0006, and PYY: F_(df=22/440)_ = 1.52, *p* < 0.0224). Time slices indicating significant group concentration differences were for GIP at 13 h (F_(df=1/97.6)_ = 9.44, *p* = 0.0028)_,_ 16 h (F_(df=1/97.6)_ = 4.77, *p* = 0.031), and 20 h F_(df=1/97.6)_ = 6.65, *p* = 0.011). For GLP-1, higher concentrations in MX trial than in XM and SED trials were seen during and following both bouts of MX exercise at time slices between 12:00 h and 15:00 h and 19:00 h and 23:00 h (F_(df=2/547)_ = 3.64 to 9.95, *p* = 0. 029 to <0.0001; F_(df=2/136)_ = 3.20 to 10.46, *p* = 0.044 to <0.0001, respectively). For PYY, MX concentrations were higher than XM concentrations at 12:00 h (F_(df=2/84.1)_ = 7.66, *p* = 0.0009), 13.00 h (F_(df=2//84.1)_ = 3.0, *p* = 0.055), and 20:00 h (F_(df=2//84.1)_ = 3.08, *p* = 0.051) time slices.

**Figure 12:**
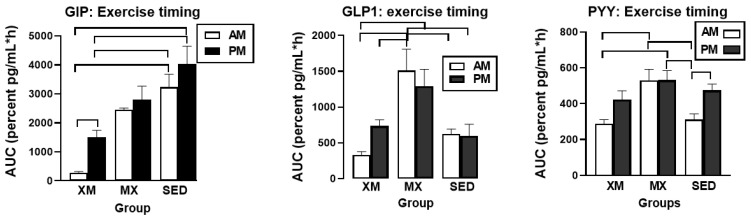
Exercise- and meal-timing effects on GIP, GLP-1, and PYY AUCs. Brackets identify significant between- and within-group differences. Morning as well as evening SED GIP AUCs were significantly higher than AM and PM XM AUCs (**left** panel). Morning MX GLP1 AUC was significantly higher than AM XM AUCs and AM and PM SED AUCs Evening MX GLP1 AUC was significantly higher than AM XM and SED AUCs (**center** panel. Morning MX PYY AUC was significantly higher than AM XM and SED AUCs (**right** panel). EX = exercise, XM = exercise before meals, MX = exercise after meals, SED = sedentary group.

GIP (F_(df=5/31.7)_ = 10.79, *p* < 0.0001), GLP-1 (F_(df=5/31.7)_ = 4.47, *p* = 0.0014), and PYY displayed significant differences in the 3-h exercise AUCs (F_(df=5/33.2)_ = 6.30, *p* = 0.0036, Figure 12) but not in the 7-h postprandial AUCs. For all group differences and for all three peptides, t values (df = 30 to 32) ranged between 3.11and 5.83, and *p* values between 0.03 and <0.0001. For GIP, both AM and PM 3-h SED AUCs were higher than the respective 3-h XM AUCs (left panel). MX AUCs were higher than either XM and SED GLP-1 (center panel) and PYY AUCs (right panel). Differences between the morning and afternoon AUCs were observed only for GIP within the XM trial (t_(df=22)_ = 4.17, *p* = 0.0004) and for PYY within the SED trial (t_(df=23)_ = 3.25, *p* = 0.029).

#### 3.5.2. Exercise- and Meal-Timing Effects on Total Ghrelin, Leptin, and CCK 

**Figure 13:**
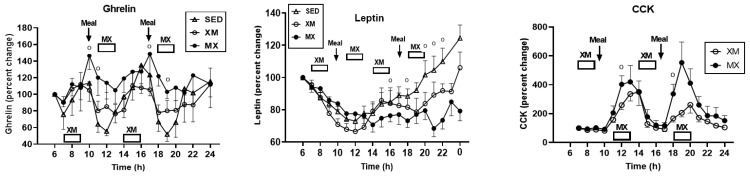
Exercise- and meal-timing effects on plasma ghrelin, leptin, and CCK concentrations. Circle superscripts identify time slices when group differences were significantly different. XM = exercise before meals, MX = exercise before meals, SED = sedentary group.

Of the three hormones, only leptin showed significant treatment effects (F_(df=2/19)_ = 4.17, *p* = 0.0314), but all three hormones had significant effects of time (ghrelin: F_(df=34/793.19)_ = 2.47, *p* < 0.0001, leptin: F_(df=19/351)_ = 24.89, *p* < 0.0001, and CCK: F_(df=26/454)_ = 19.95, *p* < 0.0001) and treatment-time interactions (ghrelin: F_(df=47/792)_ = 1.42, *p* = 0.037, leptin: F_(df=36/351)_ = 8.07, *p* < 0.0001, and CCK: F_(df=26/454)_ = 1.64, *p* = 0.0256). Group differences for ghrelin (left panel) were seen during the postprandial periods after the two XM exercise bouts between 10:00 h and 11:00 h (F_(df=2/116)_ = 3.08 and 3.26, *p* = 0.0496 and 0.418, respectively) and between 16:0 h0 and 20:00 h (F_(df=2/116)_ = 4.19 to 6.03, *p* = 0.0176 to 0.0148). CCK (right panel) displayed group differences during the MX exercise bouts with slices at 11:00 h (F_(df=1/9.7)_ = 9.61, *p*-0.0023), 18:30 h (F_(df=1/9.7)_ = 10.24, *p* = 0.0017), and 19:00 h (F_(df=1/9.7)_ = 11.03, *p* = 0.0011). Leptin (center panel) which is secreted from the stomach in addition to the subcutaneous fat tissue [44,45] produced a pattern that was different from other GIP-axis hormones. Leptin group differences were registered at 16:00 h, 18:00 h and from 20:00 to 22:00 h, when its concentrations in the two exercise groups declined while the SED concentrations continued to rise (F_(df=2/51.2)_ = 3.25, *p* = 0.047 at 16:00; F = 4.50, *p* = 0.0152 at 18:00, and F = 19.10 to 34.47, *p* < 0.0001 at 20:00 to 22:00 h) (Figure 13).

**Figure 14:**
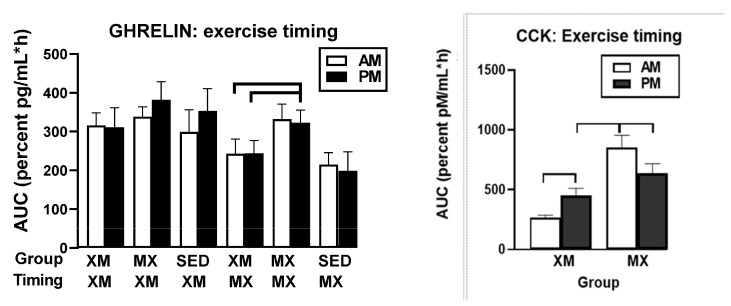
Exercise- and meal-timing effects on ghrelin and CCK AUCs. Brackets identify groups with significant between- and within-group differences. Afternoon MX ghrelin AUC was significantly higher than AM and PM XM AUCs (**left** panel). Morning MX CCK AUC was significantly higher than PM XM AUC (**right** panel). Both XM and MX CCK trials had significant within-group AUC differences. EX = exercise, XM = exercise before meals, MX-exercise after meals, SED = sedentary group.

No 3-h ghrelin AUC differences were seen between ghrelin (Figure 14, left panel) treatment groups (indicated by upper abscissa row) when the timing of exercise analysis was performed during the 3-h XM exercise period (indicated by XM in lower abscissa row). Between-group differences were significant (F_(df=5/53)_ = 8.20, *p* < 0.0001) when ghrelin 3-h AUCs were analyzed during the 3-h postprandial timing of MX exercise (indicated by MX in lower abscissa row). Higher PM XM AUCs than AM (t_(df=46.9)_ = 4.34, *p* = 0.0009) and PM (t_(df=46.9)_ = 4.39, *p*- = 0.0008) XM AUCs contributed to this effect. Significant differences in CCK AUCs (right panel, F_(df=3/23)_ = 6.83, *p* = 0.0018) were attributed to higher morning than afternoon AUCs within the MX trial (t_(df=18)_ = 3.98, *p* = 0.003) and evening XM trial (t_(df=34.7)_ = 2.82, *p* = 0.045), and to higher afternoon than morning AUCs within the XM trial (t_(df=34.7)_ = 3.02, *p* = 0.025).

## 4. Discussion

We used timing of exercise with respect to meals to determine whether the reciprocal effects of hormones and metabolites on hunger and satiation that are elicited by eating on one hand, and by moderate-intensity exercise on the other, can be used for more effective control over human appetite. The former behavior serves to increase energy gain, and the latter to increase energy expenditure. To address this issue, we examined the effects of close succession and alternation of reciprocal hormonal and metabolic effects of exercise and meal eating on the psychophysical manifestations of appetite. We provided 2 daily weight-maintenance meals preceded or followed 1 h by 2-h moderate-intensity exercise. By arranging for a meal and an exercise bout both in the morning and in the afternoon, we also could examine whether circadian timing of these behaviors in the afternoon would produce different psychophysical effects compared to the morning as suggested by greater weight loss after a big morning meal, compared to a big afternoon meal in obese women [36].

### 4.1. Testing of Testing of Hypothesis 1

**Hypothesis** **1** **(H1).** *Exercise Performed before Meals Will Increase Hunger.*

Our first hypothesis was that hormonal and metabolic responses to energy-expending exercise will increase hunger when exercise precedes the meals by one hour. Twelve hours of fasting incurred an average cumulative resting energy expenditure of 637 kcal. The energy cost of 2-h moderate-intensity XM exercise was 414 kcal (Table 4). Subjects, therefore, ate their first daily meal after a combined fasting and exercise energy deficit of 1051 kcal. This level of energy expenditure was associated with a significant increase in FFA concentrations during both exercise bouts (Figure 2) and with greater reliance on fat utilization during morning exercise (Table 4). Our results with XM exercise intensity of 45% of maximal effort lasting 2 h agreed with the data on circulating FFA concentrations and their utilization during exercise at the relative effort of between 25% and 65% lasting more than one hour [46]. In addition, increased reliance on lipid utilization during morning XM exercise also reflected hepatic glycogen depletion [24,25] evident in the rise of ketone body BHB concentration during both, but particularly the afternoon, exercise bout (Figure 3). Fuel utilization shifted in the afternoon in both XM and MX trials to predominant reliance on carbohydrates most likely due to the 485 Kcals of glucose supplied by the 10:00 h meal (Table 2 and Table 3). There was no overall treatment effect on glucose concentrations during either the XM or the MX exercise bouts, although glucose concentration reached near hypoglycemic concentrations of 70 mg/dL during the afternoon XM exercise bout (Figure 5, left panel). Glucose concentrations during both postprandial periods were only slightly higher in XM relative to other groups (Figure 5, right panel) despite significantly higher simultaneous postprandial insulin concentrations (Figure 6). The observed minor perturbations in glucose concentration during XM exercise reflected effective counter-regulation of hypoglycemic actions of insulin by glucagon, the concentrations of which rose significantly during the declines in glucose concentration in response to two MX and second XM exercise bouts (Figure 8, left panel). It would appear that increased reliance on lipid fuel during morning XM exercise, near-hypoglycemic glucose concentrations during afternoon XM exercise period, and the evidence for hepatic glycogen depletion during both XM exercise bouts, should have provided an effective stimulus for increased hunger posited by our Hypothesis 1. However, there was no treatment effect on hunger sensation in XM relative to the other two treatment groups except for a brief paradoxical suppression of hunger toward the end of second XM exercise period (Figure 9, top left panel), also reported by others [13]. A similar outcome was obtained for the desire to eat (Figure 9, top right panel) and the capacity to eat (Figure 9, lower left panel) at the 16:00 h time slice in both exercise groups. It is, therefore, reasonable to conclude that the progressive increase in hunger before the meals responded to intragastric signals associated with stomach emptying and completion of digestive and absorptive events. Absorption of the meal was completed within 5 h of insulin decline (Figure 6), and peak hunger was registered (Figure 9) during the 2 h before eating an approximately 800 kcal meal (Table 2 and Table 3). The absence of any influence on hunger of increases in circulating metabolites, a decline in glucose concentration after second XM exercise bout, of apparent depletion of liver glycogen during MX trials, or of increases during the XM exercise period in putative hunger stimulant ghrelin (Figure 13 and Figure 14, left panels), support the conclusion that the course of hunger is dependent on nutrient transit through the gastrointestinal tract and not on changes in metabolic fuels or hormone ghrelin. Additional support for this conclusion is provided by the study in which meals differing in size between 100 and 500 kcal, or a 500 kcal meal followed by 550 kcal of energy expenditure, were supplemented by intravenously delivered nutrients [47]. Only the size of caloric load of the meal ingested by mouth influenced the magnitude of hunger while metabolic and hormonal concomitants of exercise energy expenditure and parenteral supplementation were ineffective. This strongly suggests that the development of hunger depends on the size of an orally ingested meal and not on exercise-associated changes in circulating metabolites or the hormone ghrelin, and therefore Hypothesis 1 was not supported. 

### 4.2. Testing of Hypothesis 2

**Hypothesis** **2** **(H2).** *Exercise Performed after Meals Will Reduce Satiation.*

Our second hypothesis was that the sensation of fullness or satiation will decline when hormonal and metabolic effects of meal consumption that precede exercise by one hour will reduce postprandial insulin and gut hormone secretion. The rationale behind the hypothesis was that pancreatic insulin and gastrointestinal hormones CCK, GLP-1, and PYY have been implicated in stimulating the sensation of fullness or satiation in case of CCK, and satiety in case of GLP-1 and PYY, in addition to mediating digestion and absorption of meal nutrients [19,20]. As exercise inhibits insulin secretion through the sympathetic action on alpha receptors on the pancreatic beta cells [48], we also expected that it might suppress postprandial gut hormone responses as well as satiation. Insulin concentration was significantly lower during the postprandial periods in MX than in XM and SED trials, but the duration of postprandial insulin action was not altered (Figure 6). Likewise, the timing of exercise did not alter the postprandial timing of most gut peptides. None of them were secreted before the first meal, and concentrations of all increased after eating (Figure 11 and Figure 13). Postprandial levels of GLP-1 (Figure 11 and Figure 12, center panel), ghrelin (Figure 13 and Figure 14, left), and CCK (Figure 13 and Figure 14, right panel) appeared to increase rather than decline to MX exercise. GIP displayed no treatment differences during postprandial periods in all three groups (Figure 11, left panel), and increases in PYY levels in MX relative to XM and SED trials were small (Figure 11 and Figure 12 right panel). Therefore the premise of the Hypothesis 2 that exercise and exercise timing would reduce gut peptide responses to meals and thereby reduce satiation, was not supported. It should be noted that our results confirm the reports of others on acute changes in blood concentrations of gut peptides to exercise. GLP-1, PYY, [49] leptin [50], and ghrelin [51] either increased [49], decreased [48], or in the case of acylated ghrelin did not change to moderate-intensity exercise [51]. Both XM and MX exercise suppressed plasma leptin levels which continued to rise in the SED trial (Figure 13, center panel) following a postprandial pattern different from other gastrointestinal hormones. Leptin’s capacity to suppress hunger and induce weight loss in morbidly obese individuals with congenital leptin deficiency [28] was found not to serve as a putative negative feedback for hunger in individuals without such congenital abnormality [29]. While most of the research examining the role of leptin as a negative feedback over food intake has focused on the subcutaneous adipose tissue source of this hormone, it is less well known that leptin is also released from the gastric mucosa [44,45]. As a short-term postprandial hormone derived from the stomach, leptin (Figure 13, center panel), along with insulin (Figure 7) and ghrelin (Figure 13 and Figure 14, left panels) increases its secretory response not only to nutrient intake, but importantly, also to fluctuations in body energy balance [47].Thus, it appears that the sensation of fullness or satiation, like the sensation of hunger, originates in the gastrointestinal tract when nutrients are obtained by the oral route and processed by the gastrointestinal tract. Hypothesis 2 was not supported given that exercise during the immediate postprandial period increased concentrations of satiating peptides GLP-1, CCK, PYY as well as of presumed appetite stimulant ghrelin, and decreased the concentrations of the presumed appetite suppressant leptin, and none of these changes had a conspicuous effect on the sensation of fullness in the present (Figure 9), as well as in a previous, study [47].

While the hypothesis as formulated, was not supported, the timing of exercise with respect to meals did have a large and significant effect on the HOMA-IR assessment of insulin resistance (Figure 7, right panel). Compared to SED trials, HOMA-IR AUCs during XM exercise were 24 to 30% higher, and during postprandial MX exercise were 24 to 37% lower. The difference between the exacerbation of HOMA-IR during the postprandial period after XM exercise was between 42 and 56% higher than the HOMA-IR values produced by MX exercise (Figure 7, right side of right panel). This suggests that while a reduction in HOMA-IR AUCs after MX exercise is modest in the healthy postmenopausal women, insulin-resistant subjects would derive a significant health benefit by exercising at moderate intensity shortly after eating their meals. The magnitude and immediacy of HOMA-IR declines in response to 2-h moderate-intensity exercise initiated one hour after the meals, stands in contrast to the delayed reductions of HOMA-IR of similar magnitude in response to the widely-used insulin-sensitizing drug metformin [52]. It took 12 months of daily treatment with 1 g of metformin to produce a 45% reduction in HOMA-IR In insulin-resistant women with polycystic ovarian syndrome [49]. Given that this dose of metformin can produce potentially toxic lactic acidosis in individuals with even moderate renal impairment [53], the immediate benefit and the scale of a significant reduction in insulin resistance after post-meal exercise is apparent and convincing.

Our finding that 2 h of moderate-intensity postprandial exercise an hour after consuming a meal reduces insulin resistance and insulin response without having a significant effect on plasma glucose in healthy postmenopausal women is distinct from a large number of studies reporting the postprandial exercise effect principally as a way of reducing post-meal hyperglycemia. Using an exercise-and-meal timing design similar to ours, a number of studies were carried out in subjects with type 2 diabetes. In diabetics, high postprandial glycemia represents a major health problem, and reduction of glycemia with exercise during postprandial period represents a significant solution [54,55]. At least one study [56], reports a reduction in plasma glucose, but no report on plasma insulin or insulin resistance, in postmenopausal women and older men exercising at 3 METs for 15 min after three daily meals. In this study, a sustained 45-min exercise reduced blood glucose if performed during the postprandial period 2.5 h after the morning meal, but not during the postabsorptive period 4.5 h after the mid-day meal. To the best of our knowledge, our study is unique in that it shows that MX exercise after a meal reduces postprandial insulin response (Figure 6) and HOMA-IR assessment of insulin resistance (Figure 7, right panel) in healthy postmenopausal women with no significant changes in the concentration of plasma glucose. 

### 4.3. Testing of Hypothesis 3

**Hypothesis** **3** **(H3).** *Differential Timing of Exercise and Meals Will Produce Different Effects on Hunger and Satiation in the Afternoon Than in the Morning.*

Two lines of evidence make Hypothesis 3 interesting and plausible. The first one is that postprandial hyperglycemia or glucose intolerance and delayed or protracted hyperinsulinemia are prevalent in metabolically healthy individuals in the evening but not in the morning when the same carbohydrate load is delivered as either oral glucose [57,58,59,60], a carbohydrate-containing meal [61], or as intravenous glucose injection [62]. Hypoglycemic action of insulin for the same carbohydrate load also is lower in the evening than in the morning as shown by the requirement for a higher evening dose of insulin secretagogue tolbutamide [58] and insulin in type 1 diabetics [63]. A circadian influence was also implicated in the reduced β cell capacity to secrete insulin in the evening [63]. The second line of evidence is a circadian effect of timing of meals on day-long changes in glycemia or longer-term effects on body weight. Type-2 diabetic patients eating a large 700 to 850 Kcal breakfast and a small 88 Kcal dinner experienced a 20% day-long decrease in blood glucose and 11 and 30%, respectively, increases in insulin, and GLP-1 concentrations [64] compared to reversing the sizes of breakfast and dinner meals. Using the same meal strategy over 12 weeks, healthy obese women lost more body weight (10.3 vs. 3.5 kg) and reduced their waist circumference to a greater extent (8.5 vs. 3.9 cm) if they ate the larger meal in the morning [36]. These studies showed clear circadian effects on plasma glucose, insulin, and weight gain without any involvement of exercise. 

Our study was designed to compare in systematic and a structured fashion the effects of exercise and meal timing on the appetite at morning and afternoon circadian times. Our findings showed first, that the VAS appetite ratings displayed no circadian effect (Figure 10, all four panels) other than the effect of meal timing on the time course of hunger and fullness. All three hunger-associated VAS measures were low before the onset of meals and increased during the pre-meal periods, whether these were in the morning or in the afternoon (Figure 9 and Figure 10, first three panels). The pattern for fullness was reversed in that the lowest values were seen before subjects started to eat, and the highest values occurred immediately after the meal completion regardless of the time of day (Figure 9, bottom right, and Figure 10, right panels). Second, we confirmed the long-standing finding that glycemia and glucose intolerance is higher in the afternoon than in the morning not only as reported for sedentary condition [57,58,59,60] but also when exercise before eating is involved in the study (Figure 5, both panels). However, our data do not confirm that the afternoon glucose intolerance is mediated by changes in postprandial insulin response as suggested in studies that did not include exercise [62,63]. We did not find any circadian effect in insulin response during exercise timed either before or after the meals (Figure 6 and Figure 7, left panel). The absence of a circadian effect on insulin response after different timing of exercise was replicated in HOMA-IR values (Figure 7, right panel). A possible explanation for the higher afternoon compared to morning glycemia and glucose intolerance may stem from a clear circadian effect on the circulating metabolic fuels during XM exercise before the meals. Both FFA and BHB concentrations were significantly higher during the afternoon than morning XM exercise bouts (Figure 2, Figure 3 and Figure 4). They could have contributed to the observed glucose intolerance during afternoon, but not morning fasting exercise as there is evidence that the elevation of circulating FFAs [65] as well as ketones [66] during fasting induces glucose intolerance and insulin resistance.

We did not observe any consistent circadian effects in plasma concentration of hormones we measured under different timing of meals and exercise. What differences between morning and afternoon AUCs were seen were usually dependent on the absence of peptide responses before the meal onset in the morning and insufficient decline of their postprandial concentrations in the afternoon. Therefore, our Hypothesis 3 for a circadian effect on appetite associated with circadian differences in metabolic and hormonal responses as a result of timing of exercise and meals was not supported. 

## 5. Study Limitations

This study had several limitations. First, the low number of participants in the SED arm of the study precluded consistently reaching statistical significance in comparisons involving this treatment group. Second, the frequency of measurements of several gastrointestinal hormones was lower than that of circulating metabolites and insulin, again limiting the statistical significance of the results. Third, the diet offered to participants was high in carbohydrate because we followed the 2010 dietary recommendation by the US Department of Agriculture and US Department of Health and Human Services [40]. This diet may produce different postprandial and HOMA-IR effects compared to diets of different macronutrient concentrations [37]. Finally, the study was restricted to healthy non-obese postmenopausal women which precludes any generalization to the other gender, younger ages, and difference in body composition.

## 6. Conclusions

In conclusion, this study produced two significant results which apply to healthy, non-obese postmenopausal women. The main finding was that the appetite and gastro-intestinal physiology are largely independent of the increased metabolic effects of exercise performed in fasted state as well as of hormones of the gastrointestinal tract released during exercise in postprandial state. Appetite was unaffected by substantial increases in circulating metabolic fuels FFAs and BHB during exercise before the meals and by meal-associated increases in the hormones of the gastrointestinal tract that mediate digestion and absorption. There also was no correlation between the VAS measures of hunger, desire to eat, and capacity to eat and the meal-associated fluctuations in the putative hunger hormone ghrelin (Figure 9 and Figure 10, first 3 panels, Figure 13 and Figure 14, left panels). Likewise, we found no effect of meal-associated fluctuations in the putative satiation hormone CCK (Figure 13 and Figure 14, right panels) and the VAS measure of fullness (Figure 9, lower right panel, Figure 10, right panel). Instead, there was a minor, brief, and paradoxical decline in hunger and increase in fullness toward the end of first postprandial period (Figure 9), an effect that was previously reported by others [13]. The other significant finding was the contrasting effect of exercise before the meals and exercise after the meals on the homeostatic assessment of insulin resistance, HOMA-IR. The HOMA-IR measure of insulin resistance was elevated by exercise performed before eating, while exercise after the meals, relative to exercise before the meals, reduced both postprandial insulin and HOMA-IR by approximately 50% Figure 7, right panel). This finding offers a rapid and highly effective means of lowering insulin resistance in individuals with reduced insulin sensitivity that matches in magnitude the much slower effect of the anti-diabetic drug metformin. Additional finding was that VAS measures of hunger and satiation displayed no circadian effects with morning and afternoon meals and exercise. This stands in contrast to a greater elevation of circulating metabolic fuels FFAs and BHB in response to afternoon compared to morning exercise. This afternoon rise in the metabolic fuels during XM exercise may possibly explain greater afternoon carbohydrate intolerance in sedentary state as well as after exercise. Thus, temporal alternation of exercise and feeding behavior does not affect the time course or magnitude of hunger and satiation and therefore cannot be gainfully harnessed for a better appetite control. Appetite associated with meal-to-meal eating appears to respond to endogenous signals of hunger and to gastrointestinal fullness. On the other hand, timing of moderate-intensity exercise shortly after the meals can rapidly and substantially reduce insulin resistance which is exacerbated by exercise before eating. This effect matches in magnitude 12 weeks of treatment with 1 g of daily insulin-sensitizing drug metformin, but has the benefit of rapid action and no side effects.

## Figures and Tables

**Table 1:** Subject characteristics * and features of experimental manipulations.

Variable	Sedentary	Exercise before Meals (XM)	Exercise after Meals (MX)	F_(df=2,51)_, *p* T_(df=51)_, *p*
Subjects	N = 8	N = 23	N = 23	
Age (years)	55.0 ± 1.1	59.0 ± 0.8	57.6 ± 1.0	F = 2.89, *p* = 0.0619
Weight (kg)	66.1 ± 2.2	70.2 ± 2.3	68.3 ± 2.4	F = 0.4969, *p* = 0.61
BMI (kg/m^2^)	23.6 ± 0.9	25.4 ± 0.8	22.2 ± 0.9	F = 4.098, *p* = 0.023
Body fat (%)	35.1 ± 2.2	38.0 ± 1.6	36.3 ± 1.6	F = 0.548, *p* = 0.582
Fasting glucose (mg/dL)	78.0 ± 2.4	77.9 ± 3.4	78.7 ± 1.1	F = 0.048, *p* = 0.95
Fasting insulin (μU/mL)	10.6 ± 1.5	10.9 ± 1.2	10.5 ± 1.0	F = 0.148, *p* = 0.86
Fasting HOMA-IR	2.0 ± 0.2	2.0 ± 0.1	2.0 ± 0.2	F = 1.0, *p* = 0.379
Fitness level (VO2/min * kg)	25.6 ± 3.7	23.8 ± 1.8	27.7 ± 4.08	F = 1.475, *p* = 0.864
Energy intake (EI) in 2 meals (kcal)		1614.6 ± 84.9	1582.2 ± 79.7	T = 0.279, *p* = 0.785
Energy expended (EE) in 4 h of exercise (kcal)		822.3 ± 66.26	977.7 ± 116.83	T = 1.157, *p* = 0.267
Energy balance (EI-EE, kcal)		815.8 ± 88.1	604.4 ± 119.46	T = 1.424, *p* = 0.177

* All subjects were postmenopausal women.

**Table 2:** Food items, macronutrients, and calories in the morning meal.

Food Items	Wt (g)	CHO (g)	PRO (g)	FAT (g)	KCAL
Egg salad plate, multi grain bun	1 plate; 156 g	26	13	15	291
Wheat roll	0.5 each; 18.4 g	8.6	1.7	0.8	48.4
Margarine Country Crock	0.5 tub; 2.4 g			1.2	10.8
New coleslaw	85 g	11	1	3	75
Carrot sticks	1 serving; 86 g	4	1	0	20
Skim milk	1 cup; 243 g	11	8	0	76
Orange juice	0.5 carton, 63 g	7.5	0	0	30
1 banana	70 g	16	0.7	0	66.8
1 serving fresh fruit	Variable kind	15	0.8	0.2	65
Graham crackers	4 squares; 28 g	22	2	3	123
Total		121.1	28.2	23.2	806
Percent macronutrients		60.1	14.0	25.9	
Glycemic index = 58					

**Table 3:** Food items, macronutrients, and calories of the afternoon meal.

Food Items	Wt (g)	CHO (g)	PRO (g)	FAT (g)	KCAL
Bacon, cheese, & ham sandwich					
Wheat toast	2 slices; 73.4 g	28	6	2	154
Slivered ham	50 g	0	8	1.4	44.6
Bacon	1 slice; 4.7 g	0	1.5	2.7	30.3
Cheddar cheese	1 slice; 22.7 g	0	6	8	96
Tomatoes	2.5 slices; 55 g	4	0.8	0	19.2
Lettuce, green leaf, raw	14 g	0	0	0	0
Diet mayonnaise	1 pkg.; 12 g	0	0	0	0
Ketchup or mustard optional	1 pkg.; 10 g	0	0	0	0
Broccoli, cauliflower, carrots (cooked, salt can be added)	0.5 cup; 86 g	7	1	0	32
1.6 oz Tossed Greens using romaine blend	1.6 oz; 45.5 g	5	1	0	24
Diet french dressing	1 package; 12 g	2	0	0.5	12.5
1 serving fresh fruit	Variable kind	22	1.2	0.3	95.5
Cranberry Juice cocktail	0.9 carton,110 g	17	0	0	68
Rold Gold pretzels	1 oz; 28.3 g	23	2	1	109
Vanilla ice cream	57 g	13	1.8	6	113.2
Total		121	29.3	21.9	798.3
Percent macronutrients Glycemic index = 60		60.6	14.7	24.7	

**Table 4:** Characteristics and metabolic effects of exercise.

Variable	XM Exercise before Meals	MX Exercise after Meals	t_(df=19)_; *p*
Subjects	N = 10	N = 10	
Walking speed (km/h)	4.5 ± 0.1		
Walking speed (m/s)	2.0 ± 0.1		
Distance walked (km)	8.7 ± 0.2		
RMR (kcal/h)	53.1 ± 2.3	54.4 ± 4.1	t = 0.151, *p* = 0.8848
AM exercise EE (kcal/2 h)	413.7 ± 24.55	486.5 ± 57.97	t = 1.156, *p* = 0.267
Carbohydrate utilization (%) during AM exercise	43 ± 5.4	63 ± 5.8	t = 2.524, *p* = 0.024
Fat utilization (%) during AM exercise	57 ± 5.4	37 ± 5.8	t = 2.524, *p* = 0.024
PM exercise EE (kcal/2 h)	408.6 ± 24.5	491.2 ± 58.9	t = 1.295, *p* = 0.216
Carbohydrate utilization (%) during PM exercise	60 ± 3.3	67 ±3.7	t = 1.412, *p* = 0.180
Fat utilization (%) during PM exercise	40 ± 3.3	33 ± 3.7	t = 1.412, *p* = 0.180

EE = energy expenditure, RMR = resting metabolic rate, average of exercise and pos-exercise day.

## Data Availability

Data are available from the senior author.
